# Low pathogenic avian influenza virus infection retards colon microbiota diversification in two different chicken lines

**DOI:** 10.1186/s42523-021-00128-x

**Published:** 2021-09-28

**Authors:** Klaudia Chrzastek, Joy Leng, Mohammad Khalid Zakaria, Dagmara Bialy, Roberto La Ragione, Holly Shelton

**Affiliations:** 1 The Pirbright Institute, Pirbright, Woking, Surrey UK; 2 Department of Pathology and Infectious Disease, School of Veterinary Medicine, University of Surrey, Guildford, UK; 3 University of Glasgow Centre for Virus Research, Glasgow, UK

**Keywords:** Influenza virus, H9N2, Chicken microbiome, Colon microbiota, 16S rRNA gene, Metagenomics

## Abstract

**Background:**

A commensal microbiota regulates and is in turn regulated by viruses during host infection which can influence virus infectivity. In this study, analysis of colon microbiota population changes following a low pathogenicity avian influenza virus (AIV) of the H9N2 subtype infection of two different chicken breeds was conducted.

**Methods:**

Colon samples were taken from control and infected groups at various timepoints post infection. 16S rRNA sequencing on an Illumina MiSeq platform was performed on the samples and the data mapped to operational taxonomic units of bacterial using a QIIME based pipeline. Microbial community structure was then analysed in each sample by number of observed species and phylogenetic diversity of the population.

**Results:**

We found reduced microbiota alpha diversity in the acute period of AIV infection (day 2–3) in both Rhode Island Red and VALO chicken lines. From day 4 post infection a gradual increase in diversity of the colon microbiota was observed, but the diversity did not reach the same level as in uninfected chickens by day 10 post infection, suggesting that AIV infection retards the natural accumulation of colon microbiota diversity, which may further influence chicken health following recovery from infection. Beta diversity analysis indicated a bacterial species diversity difference between the chicken lines during and following acute influenza infection but at phylum and bacterial order level the colon microbiota dysbiosis was similar in the two different chicken breeds.

**Conclusion:**

Our data suggest that H9N2 influenza A virus impacts the chicken colon microbiota in a predictable way that could be targeted via intervention to protect or mitigate disease.

**Supplementary Information:**

The online version contains supplementary material available at 10.1186/s42523-021-00128-x.

## Introduction

Avian influenza A viruses (AIV), belong to the Orthomyxoviridae family, have segmented, single-stranded, negative sense RNA genomes with enveloped virions [1]. Based on pathogenicity, AIV can be categorised as low and high pathogenicity AIVs. Among low pathogenicity AIV (LPAIV), the H9 subtype circulates globally in wild birds and is endemic in domestic poultry in many countries in the Middle Eastern, Africa and Asia [2–8]. The majority, approximately 75%, of natural H9 isolates, are paired with the N2 neuraminidase (NA) subtype and are most frequently isolated from chickens, followed by waterfowl, pigeons, quail, and turkeys [9]. Infected chicken flocks usually experience mild respiratory distress, diarrhoea, decreased body weight in broilers, and a drop in egg production in layer hen flocks, the mortality rates are generally low, below 20% [10–14]. However, infected poultry are more susceptible to secondary infections, including bacterial infection and in such cases the mortality rate can increase up to 65% [15–18]. It has been shown that certain bacteria, such as *Staphylococcus* spp. can enhance influenza virus activation by indirect proteolytic cleavage and activation of the hemagglutinin (HA) using the virulence factor, staphylokinase [19].

The interplay between pathogens and host microbiota play an important role in health and disease in many vertebrates [25–28]. Compelling evidence has shown that the gut microbiota can play a role in pathogenesis of various human diseases including those with primary involvement outside of the gut, such as respiratory, renal, or neurologic [20–22]. For instance, recent studies reveal that immune protection and severity of infection by gammaherpesvirus, which can cause severe vasculitis and lethal pneumonia or respiratory syncytial virus infection of the lungs, can be dependent on the profile of the human gut microbiota [23, 24].

Studies in the chicken model have shown compositional changes in gut microbiota and differences in some of immune gene expression levels following H9N2 avian influenza virus (AIV), Newcastle disease virus (NDV) or infectious bronchitis virus (IBV) infection [29–32]. However, there are many environmental factors, such as age, breed, diet, housing, hygiene, and temperature that can also affect chicken microbiota [33, 34] and thus might change the interactions between the host and viruses during the time of infection. Furthermore, a recent murine study, demonstrated how polymorphism in host genes shape the intestinal microbiota and how host genetics influence the output microbiota by comparing genetically identical and genetically diverse mouse models [35]. It has been shown in chicken model that genetic background can influence viral pathogenesis [36–40]. For instance, two different inbred chicken lines, Fayoumi and Leghorn, have been used to evaluate mechanisms of genetic response to several different pathogens. [41, 42]. Deist et al. [38] showed that Fayoumis chickens infected with NDV had a faster viral clearance than Leghorns chickens and higher serum antibody levels.

There are many factors contributing to the complex interplay between pathogens and host microbiota. Understanding of changes in host microbiota resulting from viral infections is particularly of interest, as it could provide information for developing new methods for infectious disease prevention and treatment. In this study we used two different chicken breeds, Rhode Island Red and VALO leghorns, reared in different facilities, to assess colon microbiota changes following H9N2 AIV infection. We analysed the commonality and differences in the host microbiota changes during and following infection of these two chicken lines.

## Materials and methods

### In vivo chicken study design

Two separate in vivo experiments were performed. In Experiment 1, we used specific pathogen free (SPF) Rhode Island Red (RIR) chickens to assess the effect on H9N2 infection on RIR host colon microbiota. A total of 45 one-day-old SPF RIR chickens were host in pens until 2-weeks of age when chickens were randomly allocated into two experimental groups: control (n = 25) and H9N2 challenged (n = 20). Five control group birds were culled before the challenge at 3-weeks of age to establish the starting microbiota profile. Colon samples were collected at day 2 (n = 10), day 4 (n = 10) and day 10 (n = 20) post challenge from both infected and control groups. In Experiment 2, SPF RIR were compared to SPF VALO chickens to assess differences in host microbiota following H9N2 infection in the two different chicken breeds. A total of 104 one-day-old SPF chickens (n = 52, RIR and n = 52, VALO) were host separately in two pens. Colon samples were collected from each breed control group (n = 5, RIR and n = 5, VALO) at day 7, 14, 21, 28 and 32 of age. At 3 weeks of age, 18 birds from each group were randomly selected and H9N2 challenged. The colon samples from challenged birds were collected before the challenge (day 0), at day 3 post-challenge and day 14 post-challenge.

RIR chickens were provided as day old chicks from the National Avian Resource Facility (NARF) located at The Roslin Institute, Edinburgh, UK whilst VALO chickens were delivered as fertilised eggs from VALO BioMedia GmbH (Germany) which were set and hatched at the Biological Service Unit (Poultry) at The Pirbright Institute (UK). The feed was provided ad libitum according to manufacture instruction for the chicken age. Both RIR and VALO chicks move from starter feed to grower at 3 weeks old. In both experiments. Control groups were housed in raised floor pens whilst AIV challenged chickens were housed in self-contained BioFlex® B50 Rigid Body Poultry isolators (Bell Isolation Systems) maintained at negative pressure. The H9N2 challenged birds received 100 µl of 10^4^ pfu (50 µl in each nare) of H9N2 AIV, A/chicken/Pakistan/UDL01/08. Blood samples were taken from a wing vein pre-challenge and at day 14 post-challenge for serum collection. All birds were swabbed daily from day of challenge until 8 days post infection in both cloacal and buccal cavities to determine viral shedding. Swabbing was carried out with sterile polyester tipped swabs (Fisher Scientific, UK) which were transferred into viral transport media (Who 2006), vortexed briefly, clarified by centrifugation and stored at − 80 °C prior to virus detection. At appropriate timepoints chickens were humanely euthanized either by intravenous administration of sodium pentobarbital if housed in isolators or by cervical dislocation in the case of the control groups. All colon samples were collected from the distal part of colon (2-cm sections of each chicken), and then snap-frozen. Samples were stored at − 80 °C until subsequent analysis. Body weights were monitored daily until the end of experiment.

### Virus and cells

Recombinant A/chicken/Pakistan/UDL01/08 H9N2 virus was generated using reverse genetics as previously described [66]. Virus stocks were produced via passage in 10 day old embryonated chicken eggs; the allantoic fluid harvested after 48 h and titrated by plaque assay on MDCK cells (ATCC).

Madin-Darby Canine Kidney (MDCK) cells (ATCC) were maintained in DMEM (Gibco-Invitrogen, Inc.) supplemented with 10% foetal bovine serum (Biosera, Inc.), 1% penicillin/streptomycin (Sigma-Aldrich, Inc.) and 1% non-essential aa (Sigma-Aldrich, Inc.).

### Serology

Haemagglutinin inhibition (HI) assays were carried out using challenge virus A/Chicken/Pakistan/UDL01/08(H9N2) antigen. HI assays were performed according to standard procedures [68]. Titres were expressed as log2 geometric mean titres (GMT). Samples with titres below 3 log2 GMT were considered negative.

### Virus shedding

Buccal swab samples from both challenge experiments were titrated by plaque assay on MDCK cells. MDCKs were inoculated with tenfold serially diluted samples and overlaid with 0.6% agarose (Oxoid) in supplemented DMEM (1 × MEM, 0.21% BSA V, 1 mM l-Glutamate, 0.15% Sodium Bicarbonate, 10 mM Hepes, 1 × Penicillin/Streptomycin (all Gibco) and 0.01% Dextran DEAE (Sigma-Aldrich, Inc.), with 2 µg/ml TPCK trypsin (SIGMA). They were then incubated at 37 °C for 72 h. Plaques were developed using crystal violet stain containing methanol. Viral titres were expressed as log10 plaque forming units (PFU) per ml and the limit of detection is 0.9 log10 PFU per ml for this assay.

Cloacal swab samples from both challenge experiments were titrated by qRT-PCR assay for the viral matrix (M) protein. qRT-PCR analysis was completed using the Superscript III Platinum one-step qRT-PCR kit (Life Technologies). Cycling conditions were: (1) 5-min at 50 °C, (2) a 2-min step at 95 °C, and (3) 40 cycles of 3 s at 95 °C, 30 s of annealing and extension at 60 °C. Cycle threshold (CT) values were obtained using 7500 software v2.3. Mean CT values were calculated from triplicate data. Within viral M segment qRT-PCR, an M segment RNA standard curve was completed alongside the samples to quantify the amount of M gene RNA within the sample from the CT value. T7 RNA polymerase-derived transcripts from UDL-01 segment 7 were used for preparation of the standard curve.

### DNA extraction and 16S rRNA gene amplification

Samples were extracted in batches (experiment 1, one batch and experiment 2, two batches). DNA was extracted using the PowerSoil® DNA Isolation Kit (Mo Bio) according to manufacturer instruction. Controls for DNA extraction reagents only were included for each batch of DNA extractions along with ZymoBIOMICS Microbial Community Standards (Zymo Research) and E. coli DH5α (ThermoFisher). The V2–V3 region of the 16S rRNA gene was amplified via PCR as described previously by Glendinning et al. [51]. Briefly, a nested PCR protocol was performed using the V1–V4 primers 28F (‘5–175 GAGTTTGATCNTGGCTCAG-3’) and 805R (‘5-GACTACCAGGGTATCTAATC-3’) followed by the V2-V3 primers 104F (‘5-GGCGVACGGGTGAGTAA-3’) and 519R (‘5–177 GTNTTACNGCGGCKGCTG-3’) with Illumina adaptor sequences and barcodes.

### Sequencing and data analysis

Libraries were analysed on a High Sensitivity DNA Chip on the Bioanalyzer (Agilent Technologies) and Qubit dsDNA HS assay (Invitrogen). The amplicon libraries were pooled in equimolar concentrations, before loading on the flow cell of the 500 cycle MiSeq Reagent Kit v2 (Illumina, USA) and pair-end sequencing (2 × 250 bp). The amplicon-based sequencing was performed using the Illumina MiSeq platform at The Pirbright Institute. Bioinformatic analysis was implemented using the Quantitative Insights into Microbial Ecology (QIIME) platform version qiime2-2019.10. Low-quality sequencing reads were quality trimmed and denoise using DADA2. Potential chimeric sequences were removed using UCHIME, and the remaining reads assigned to 16S rRNA operational taxonomic units (OTUs) based on 97% nucleotide similarity with the UCLUST algorithm and then classified taxonomically using the SILVA reference database (silva-132-99-nb-classifier). Taxonomy was then collapsed to the genus-level. The microbial community structure was estimated by microbial biodiversity (i.e., species richness and between-sample diversity). Shannon index, phylogenetic diversity, and the observed number of species were used to evaluate alpha diversity, and the unweighted UniFrac distances were used to evaluate beta diversity. All these indices (alpha and beta diversity) were calculated by the QIIME pipeline. Data was visualized using R package “ggplot2” ver 3.2.1 [67]. All raw data used in the analysis are available as fasta files at https://figshare.com/account/home#/projects/114048.

### Statistical analysis

Kruskal Wallis pairwise statistics were used to assess differences in community richness (Shannon diversity, phylogenetic diversity, and the observed number of species, OTU). In addition, Spearman correlation coefficient and simple linear regression was used to evaluate temporal changes in community richness that occurred during AIV infection between control and infected birds. A multivariate ANOVA (PERMANOVA) analysis was used to determine significant differences in β diversity distances across groups. Principal-coordinate analysis (PCoA) graphs were constructed to visualize similarity between the samples over the time of AIV infection. Additionally, Principal Component Analysis (PCA) was performed using OTU matrix. The Linear Discriminant Analysis Effect Size (LEfSe) algorithm and analysis of composition of microbiotas (ANCOME) were used to identify differentially abundant taxa between the groups. For LEfSe analysis, depends on the experiments, different groups were assigned as comparison classes and were analysed by days. Briefly, in experiment 1, RIR control and RIR AIV infected groups were assigned as comparison classes and assessed at day 0, day 2, day 4 and day 10 post-challenge. In experiment 2, RIR control and VALO control groups were assigned as comparison classes and analysed at 7, 14, 21, 28 and 32 day of age whilst RIR and VALO AIV infected groups that represented separate classes were analysed at day 0, day 3 and day 14 post-challenge. LEfSe identified features that were statistically different between assigned groups and then compared the features using the non-parametric factorial Kruskal–Wallis sum-rank test (alpha value of 0.05) and Linear Discriminant Analysis (LDA) > 2.0.

## Results

### H9N2 AIV infection of chickens causes mild clinical signs

In both of our experiments, all birds survived H9N2 AIV challenge. RIR and VALO H9N2 infected chickens showed mild lethargy and diarrhoea especially between day 2 and day 4 post-challenge. There were no significant differences in body weight between control and infected birds for either breed (Additional file 1: Fig. S1). There were significant differences in body weight between the RIR and VALO chickens. At day 0 the RIR control group were on average 54.31 g (± 5.5 g SEM) heavier than the VALO control group (*p* < 0.0001) whereas at day 14 the RIR control group was 180.3 g (± 38.2 g SEM) heavier than the VALO control group (*p* = 0.0421) (Additional file 1: Fig. S1).

### VALO chickens shed H9N2 virus infection from the buccal cavity, a day longer than RIR chickens

**Fig. 1:**
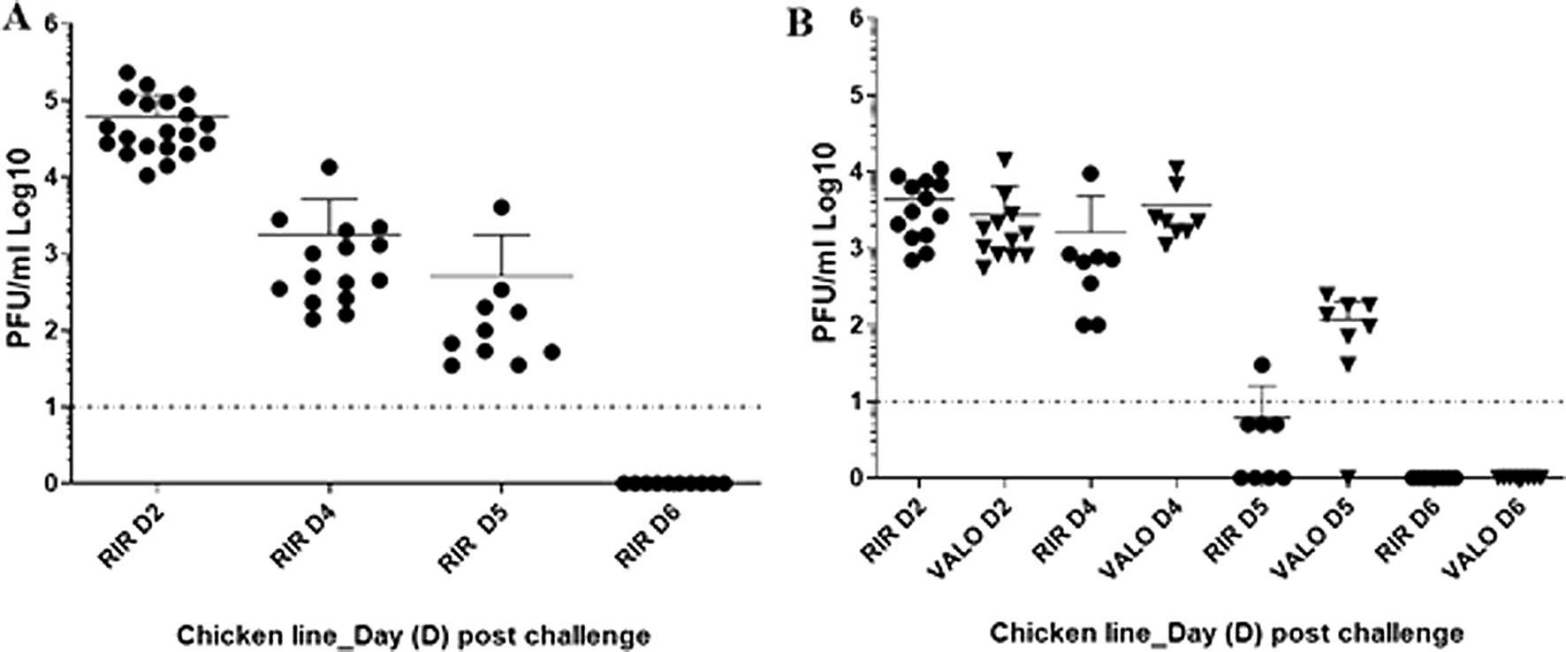
Oropharyngeal virus shedding from chickens after challenged with H9N2 AIV A/chicken/Pakistan/UDL01/08. **a** Viral titre recovered from oral swabs at day 2 (D2), 4 (D4), 5 (D5), 6 (D6) post AIV challenge from SPF RIR chickens (Experiment 1). **b** Viral titre recovered from oral swabs at day 2 (D2), 4 (D4), 5 (D5), 6 (D6) post challenge from SPF RIR and SPF VALO chickens (Experiment 2). Viral titres are expressed as log10 plaque forming units (PFU) per ml. A dashed line indicates the lower limit of detection for the plaque assay carried out on MDCK cells, 0.9 log10 PFU per ml

Buccal viral shedding was determined by testing oropharyngeal (OP) swabs at day two, day three, day four, day five and day six post-challenge by plaque assay on MDCK cells (Fig. 1). In experiment 1 where only RIR chickens were infected, all chickens shed virus from the OP cavity on day two with the average titre shed being 6.2 × 10^4^ pfu/ ml (± 12,730 pfu/ml SEM). Virus titre declined on day four and day five, with no virus being recovered from samples taken on day six post infection (Fig. 1a). In experiment 2, both RIR and VALO chickens were challenged with the same dose of the same H9N2 virus. At day two post challenge, all chickens in both lines shed virus from the OP cavity with no statistical difference in titre shed between the chicken lines being observed (average titres being 4.3 × 10^3^ pfu/ ml (± 921 pfu/ ml SEM) for RIR and 2.7 × 10^3^ pfu/ml (± 1086 pfu/ml SEM) for VALO). Similarly, to experiment 1 virus titres in the OP cavity declined on days four and five with no observed virus shedding on day six post challenge for either line. We did see differences in the rate of viral clearance between the RIR and VALO chicken lines from day four post challenge onwards. On day four the mean OP virus shed was 1.6 × 10^3^ pfu/ml (± 1129 pfu/ml SEM) for the RIR compared to 3.6 × 10^3^ pfu/ ml (± 1121 pfu/ml SEM) for the VALO (*p* value = 0.2465). On day five only 1 out of 8 RIR birds shed virus to titres above the limit of detection for the plaque assay whereas 7 out of 8 VALO birds did (average virus shed was 1.3 × 10^2^ pfu/ml) (Fig. 1b). Cloacal viral shedding of virus by all the birds in both experiments was conducted by qRT-PCR for viral M gene, but only a single RIR bird on day four post challenge had a positive result (data not shown).

Antibody responses to the H9N2 virus prior to challenge and at 10 days (Experiment 1) and 14 days (Experiment 2) post-challenge were measured by Hemagglutination inhibition (HI) assay. All infected birds in both experiments seroconverted with an average HI titre above 8 log_2_ (Additional file 1: Fig. S2). No significant differences between RIR and VALO infected birds were found.

### Batch processing of colon tissue from chickens did not affect the median number of operational taxonomic units (OTUs) obtained from 16S rRNA amplicon sequencing

Following euthanasia of chickens at various time-points colon samples were aseptically sampled at *post-mortem* examination, homogenised and the microbial DNA extracted. The bacterial DNA was processed to give 16S rRNA amplicons and subjected to Illumina Miseq sequencing. We performed a pre-processing and quality check on the raw reads obtained from the Illumina Miseq platform, which removed about 20% of the sequences. All sequences were then normalized to 62,000 sequences per sample resulting in a total number of 4,094,860 OTUs with a median of 119,082.000 OTUs per sample in Experiment 1 (Batch 1), 5,970,048 of OTUs with a median of 156,783 OTUs per sample in H9N2 infected groups (Batch 2) and 8,313,621 of OTUs with a median of 173,833.5 per sample in control groups in Experiment 2 (Batch 3) (Additional file 1: Fig. S3). This raw data was then used in our described pipelines (“Material and methods” section) to analyse changes in the biodiversity of the chicken colon microbiota following H9N2 infection and whether chicken breed impacted these changes.

### Microbiota alpha diversity indices increased over time in healthy chicken colon

**Fig. 2:**
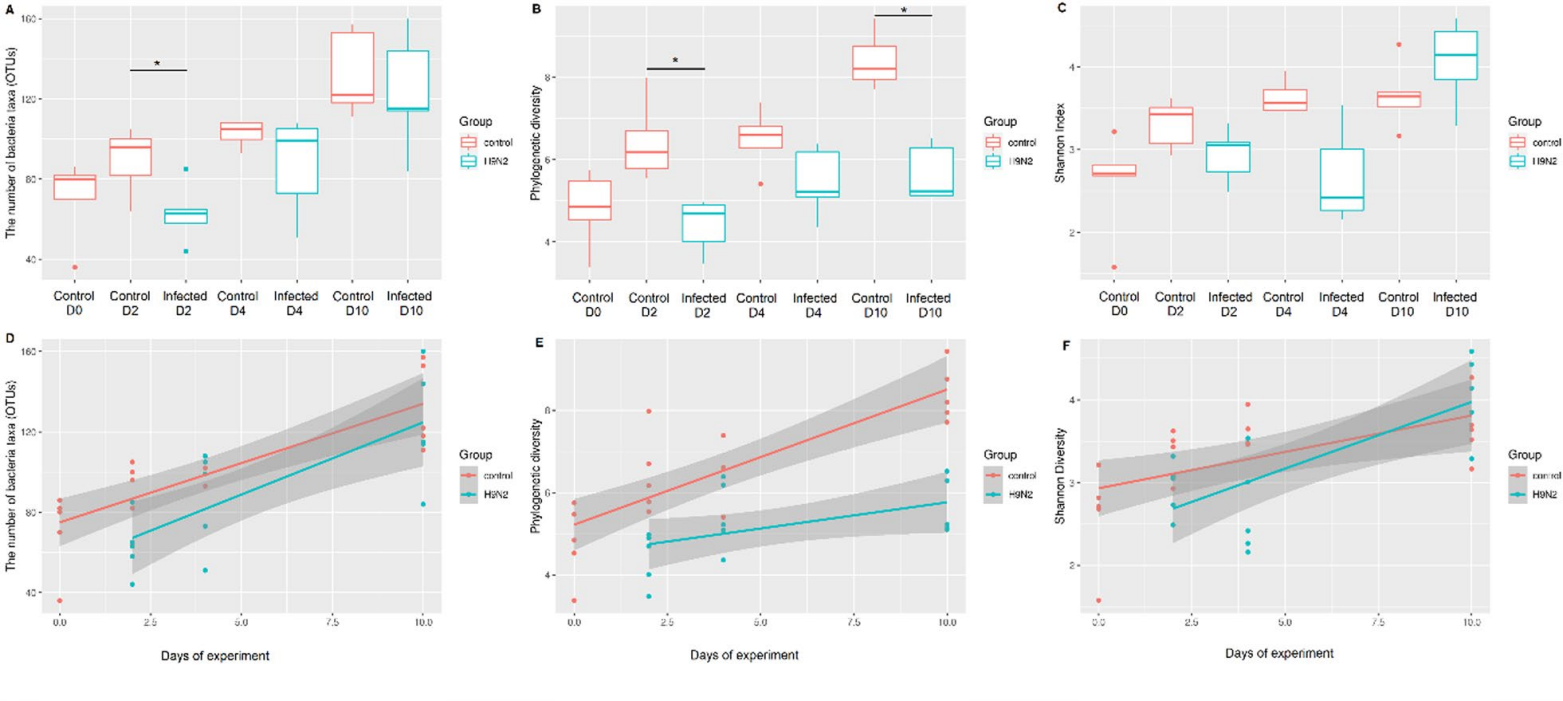
Chicken colon microbiota alpha diversity indices and simple linear regression plots comparing H9N2 infected and uninfected RIR chickens. SPF RIR chickens were challenged with H9N2 AIV A/chicken/Pakistan/UDL01/08. Colon samples were collected at day 0 (pre-challenge), day two, day four and day ten post challenge from H9N2 infected group and non-infected control groups. **a** The number of observed bacteria taxa (OTUs) in different experimental groups. **b** Faith's phylogenetic diversity in different experimental groups. **c** Shannon indices in the different groups. Kruskal Wallis pairwise statistics were used to assess differences in community richness; *p ≤ 0.05. Only statistical differences between the groups at each time point are marked on the graph. The bottom row of linear regression plots shows the change in relative abundance (**d**), phylogenetic diversity (**e**), Shannon index (**f**) from time t to time t + 1 (y-axis) in H9N2 AIV A/chicken/Pakistan/UDL01/08 infected and control groups

**Fig. 3:**
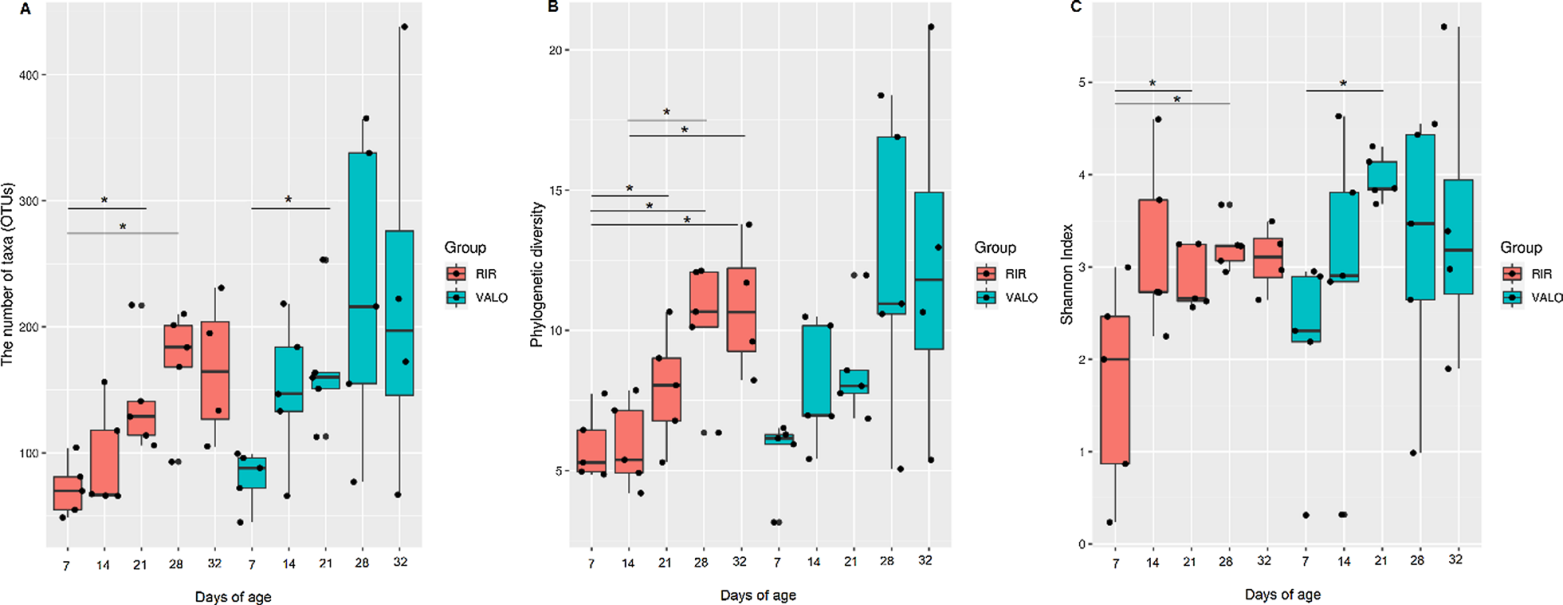
Temporal changes in RIR and VALO chicken’s colon microbiota. **a** The number of observed bacteria taxa (OTUs) at different time points. **b** Faith's phylogenetic diversity at different time. **c** Shannon indices at different time points. Kruskal Wallis pairwise statistics were used to assess differences in community richness; *p ≤ 0.05. Colon samples were collected at day 7 of age, day 14, day 21, day 28 and day 32 of age. Major changes in alpha diversity indices were seen in 14 days interval, especially between day 7 and day 21 of age

A temporal change in diversity measure, number of OTUs, phylogenetic diversity and Shannon diversity, were observed in control groups in both experiments (Figs. 2 and 3 and Additional file 1: Fig. S4). When RIR and VALO chicken colon microbiota composition was analysed weekly (Experiment 2), we identified that statistically significant changes in alpha diversity indices in control groups occur in the time frame of 14 days, especially between day 7 and day 21 of age, suggesting that a 2 weeks’ period was required to see significant maturity changes in healthy colon microbiota (Additional file 2: Table S1). Kruskal Wallis statistical testing showed a statistically significant temporal changes in number of OTUs (the number of bacterial species detected in a sample, species richness) between day 7 and day 21, day 7 and day 28 and day 7 and 32 of age in RIR chickens and between day 7 and day 21, day 7 and day 28 of age in VALO chickens (Additional file 2: Table S1). Similarly, phylogenetic significant changes were seen between day 7 and day 21, day 7 and day 28 and day 7 and 32 of age and day 14 and day 28 in RIR chickens And for VALO chicken between day 7 and day 21 of age (Additional file 2: Table S1). Shannon index, a measure of species distribution in a sample, significantly increased between day 7 and day 21 of age in both chicken breeds suggesting a more even community evolves over time (Additional file 2: Table S1). Spearman correlation coefficient (rs) between time and number of detected OTUs was 0.5968 (*p* < 0.0001) and time and phylogenetic diversity was 0.6544 (*p* < 0.0001) in control RIR and VALO groups.

The association between time and microbial diversity was also tested via simple linear regression (Additional file 2: Table S2), with microbial alpha diversity (the number of OTUs and phylogenetic diversity metrices) used as the dependent variable (Additional file 1: Fig. S4). In control groups, the number of OTUs, phylogenetic diversity and Shannon index significantly correlated with time (*p* > 0.001) (Additional file 1: Fig. S4). However, individual variation within the colon microbiota, especially VALO line at later time points (day 28 and day 32 of age) was greater as compared to age of 7-or 14-days-old.

### Colon microbiota alpha diversity indices are significantly lower during the acute phase of H9N2 infection in chickens which is not altered in two different chicken breeds

First alpha diversity was quantified by the total number of observed species (OTUs) in each sample in experiment 1 (H9N2 infected versus sham infected RIR chickens) (Fig. 2a). We observed a significant reduction (*p* < 0.05 as determined Kruskal Wallis testing), in OTU number in colon samples from chickens at 2 days post influenza virus challenge (Fig. 2a and Additional file 2: Table S3), which corresponds with high levels of viral shedding (Fig. 1a), compared to the sham infected group at the same timepoint. The OTU numbers at day four and ten post challenge were not significantly different to the control group however, we observed higher variability in interquartile range of OTU numbers with the infected groups at days four and ten compared to the corresponding control groups and compared to the day 2 groups. This suggests that the acute reduction in number of OTU on day two occurred more uniformly than the recovery of OTU numbers post infection in individuals (Fig. 2a). Faith`s phylogenetic index of observed species which describes OTU diversity and Shannon diversity index which indicates OTU evenness were also measured (Fig. 2b, c). Kruskal Wallis statistical testing showed statistically significant (*p* < 0.01) reduced phylogenetic alpha diversity in H9N2 infected RIR chicken at acute phase of infection (day 2 post-challenge) and at day 10 post challenge as compared to control groups at the same time point measured (Fig. 2b and Additional file 2: Table S3). We observed no statistical difference in the Shannon Index between any of the infected groups and associated controls (Fig. 2c and Additional file 2: Table S3). Simple linear regression analysis showed that the Shannon index and the number of OTUs increased over the course of H9N2 infection in infected RIR group, similarly to the control group (Fig. 2d, f and Additional file 2: Table S2). The number of OTUs significantly correlated with time, as determined by linear regression (R2 = 0.666, *p* < 0.0001, equation Y = 5.914*X + 74.92 for control RIR chickens, and R2 = 0.510, *p* = 0.0004, equation Y = 5.914*X + 62.44 for H9N2 infected RIR chickens) (Fig. 2d and Additional file 2: Table S2). The Faith`s phylogenetic diversity significantly correlated with time for both the control and infected group but the increase in phylogenetic diversity of the infected group was retarded as compared to the control group, suggesting that diversity development of the chicken colon microbiota was reduced by H9N2 virus infection (R^2^ = 0.6894, *p* < 0.000, equation Y = 0.3286*X + 5.220 for control group, and R^2^ = 0.2114, *p* = 0.0414, equation Y = 0.1057*X + 4.653 for the H9N2 infected group) (Fig. 2e and Additional file 2: Table S2).

**Fig. 4:**
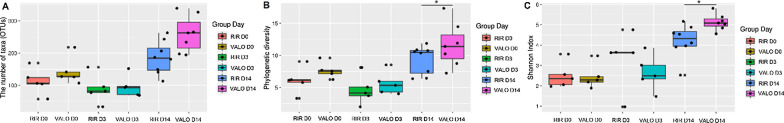
Alpha diversity indices of colon microbiota compared for two divergent chicken lines, RIR and VALO. **a** The number of observed bacteria taxa (OTUs) at different time points post infection. **b** Faith's phylogenetic diversity at different time points post infection. **c** Shannon indices at different time points post infection. Colon samples were collected pre-challenge, at day 0 of experiment, (D0) and day 3 (D3) and day 14 post challenge (D14). Chickens were challenge with recombinant A/chicken/Pakistan/UDL01/08 H9N2 LPAIV. Kruskal Wallis pairwise statistics were used to assess differences in community richness; * *p* ≤ 0.05

Figure 4 shows the alpha diversity measurements compared for RIR and VALO chicken breeds infected with H9N2 at day zero, day three and day fourteen post challenge. As it was seen in experiment 1, the number of OTUs and phylogenetic diversity dropped during the acute phase of infection (day 3 post infection) following H9N2 AIV challenge in both chicken lines (Fig. 4a, b) however this was not statistically significant (Additional file 2: Table S3). Between the chicken breeds no statistically significant differences in alpha diversity metrices before the challenge (day zero, D0) and during the acute phase of infection (day 3 post-challenge) (Fig. 4) were observed, both lines responded in a similar fashion to infection by H9N2 AIV. Interestingly, an increased Faith`s phylogenetic (Fig. 4b) and Shannon diversity (Fig. 4c) indices were found in VALO chickens as compared to RIR chickens at recovery phase of H9N2 infection (day 14 post-challenge) (*p* < 0.05) (Additional file 2: Table S3).

### Beta diversity gut community changes are associated with H9N2 AIV infection and chicken breed

**Fig. 5:**
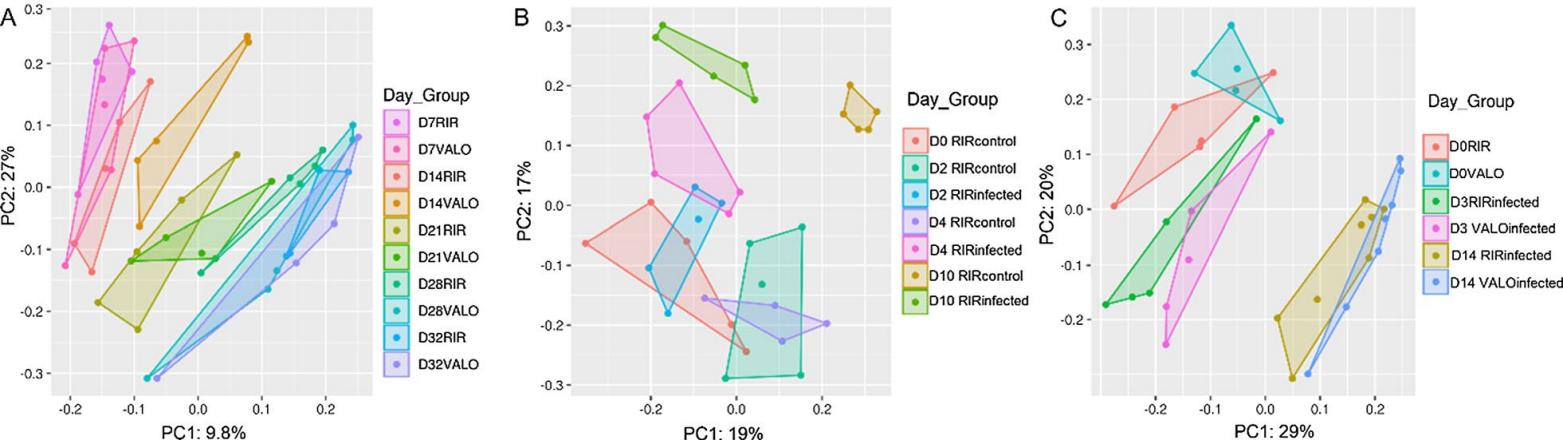
Compositional PCA plots of chicken colon microbiota using unweighted UniFrac distance data, categorized by time point and group (Day_Group). **a** PCA analysis of RIR and VALO chicken colon microbiota grouped by day. The samples were collected at day 7 of age (D7), day 14 (D14), day 21 (D21), day 28 (D28) and day 32 of age (D32). **b** PCA analysis of H9N2 infected and uninfected, control RIR chickens at day 0 (D0) pre-challenge, day 2 (D2), day 4 (D4) and day 10 post-challenge (D10). Chickens were challenged with recombinant A/chicken/Pakistan/UDL01/08 H9N2 LPAIV at D0 of experiment. **c** PCA analysis of H9N2 infected RIR and VALO chickens at day 0 pre-challenge (D0), day 3 (D3) and day 14 (D14) post-challenge. Chickens were challenge with recombinant A/chicken/Pakistan/UDL01/08 H9N2 LPAIV at D0 of experiment. PC1, PC2; percent variables explained (%)

To compare the beta diversity among the groups at different time points, we performed Principal Coordinates Analysis (PCoA) and Principal component analysis (PCA) using the unweighted Unifrac data of taxonomic composition that includes phylogenetic diversity metrics (Fig. 5). A significant separation in the control groups was observed over the time of birds’ maturity (Fig. 5a). Significant differences in beta diversity within the RIR and VALO breed control groups were found in time-based manner of at least 7 days interval. Analysis of variance have shown significant differences between day 7 and 14, day 21 and 32, day 14 and 21, day 28 and 32 but not between day 28 and 32 in both chicken breed control groups (Additional file 2: Table S4). The only statistically significant difference between RIR and VALO chicken breeds was seen at day 21 of age for the control groups (Fig. 5a and Additional file 2: Table S4).

PCoA plots indicate a significant separation between control and H9N2 RIR infected chickens at all time points tested (day two, day four, and day ten post challenge) for experiment 1 (Fig. 5b). Analysis of variance (PERMANOVA) for measuring beta-diversity showed that the H9N2 RIR infected group had significantly lower diversity as compared to RIR control group at all time points tested (Additional file 2: Table S4). Similarly, significant separation in beta diversity was observed between day zero and day three post challenge for both RIR and VALO H9N2 infected chickens (Fig. 5c). Analysis of variance showed significant lower diversity in RIR and VALO infected groups as compared to sham infected groups at the same time point tested (Additional file 2: Table S4).

### Bacterial taxa associated with H9N2 infection

**Fig. 6:**
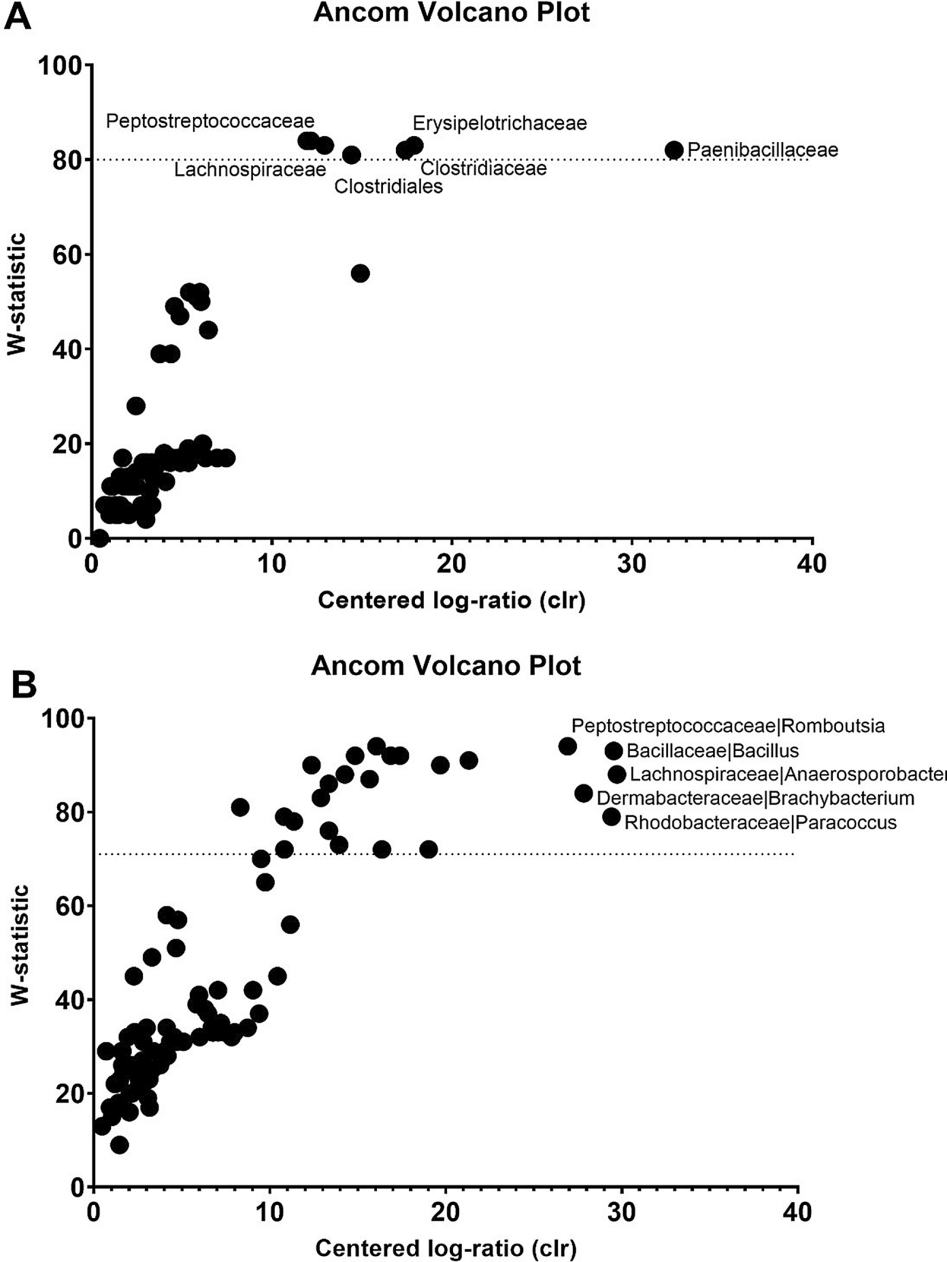
Volcano plot for the analysis of composition of microbiotas (ANCOM) test. Significant bacterial taxa are above the line. Taxa on the top-left corner are distinct species but with small proportions, (low f-score). Truly different taxa are depicted as one moves towards the far right (high W-statistic). **a** ANCOM test applied to control RIR and H9N2 infected RIR chickens (Experiment 1). **b** ANCOM test applied to RIR and VALO H9N2 infected chickens (Experiment 2)

**Fig. 7:**
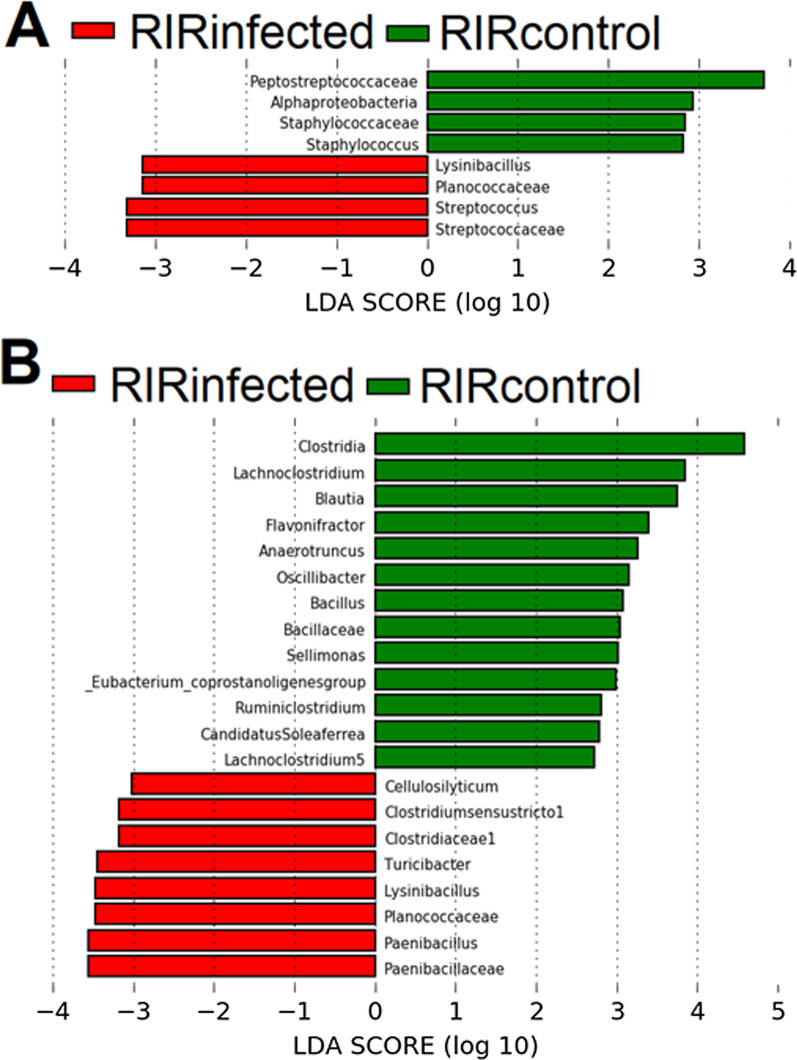
Linear discriminant analysis Effect Size (LEfSe) analysis identifying taxonomic differences in the colon microbiota of RIR H9N2 AIV infected and control chickens. (Histogram of LDA scores of 16S rRNA gene sequences in H9N2 infected chickens at day 2 (**a**) and at day 4 post challenge (**b**) and respective control groups. LDA scores (log_10_) above 3.0 and *p* < 0.05 are shown. Chickens were challenge with A/chicken/Pakistan/UDL01/08 H9N2 LPAIV. Infected groups enriched taxa are indicated with a positive LDA score (green), and taxa enriched in the control groups are characterized by negative score (red)

**Fig. 8:**
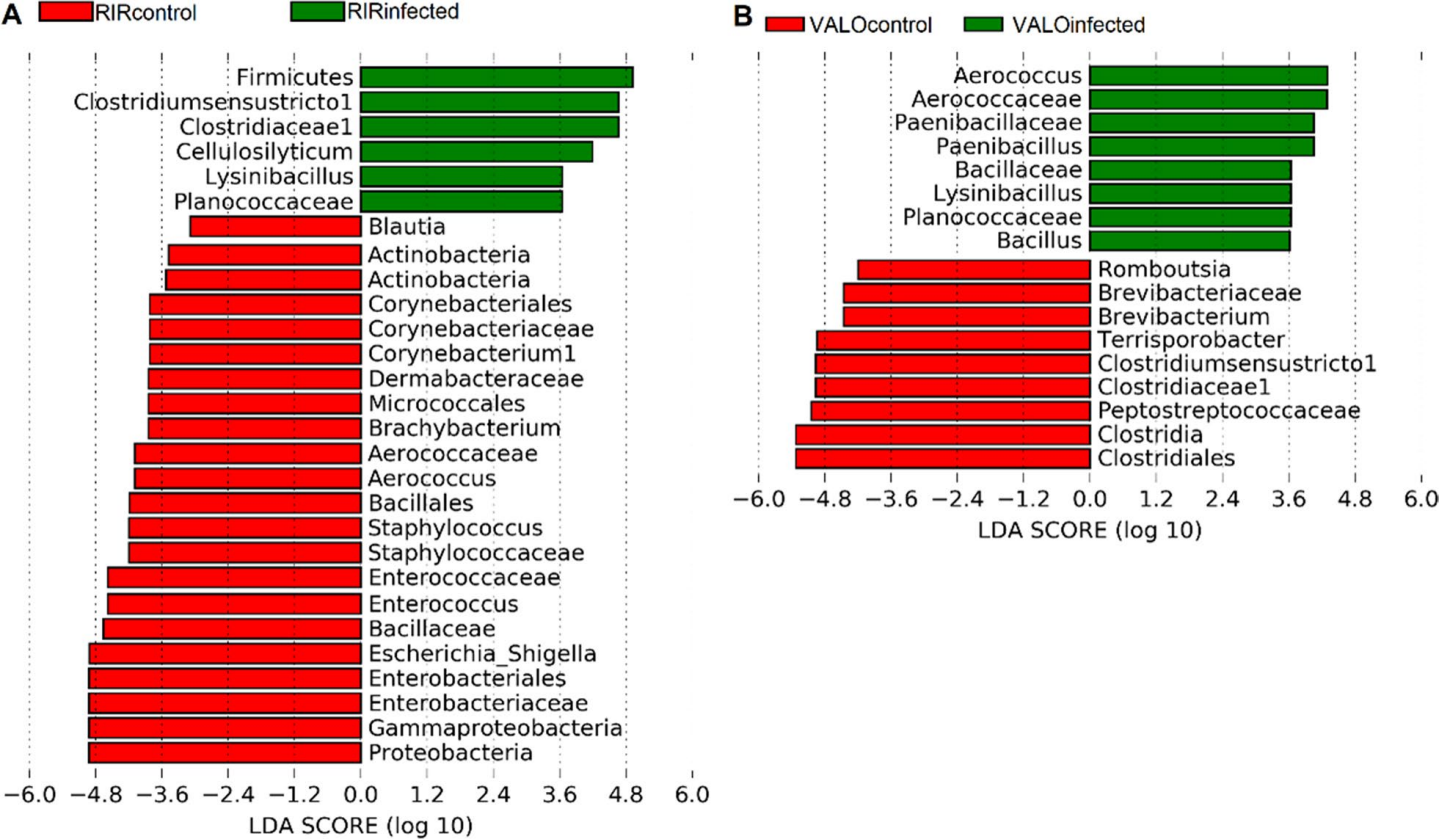
Linear discriminant analysis effect size (LEfSe) analysis identifying taxonomic differences in the colon microbiota of H9N2 AIV infected and control RIR and VALO chickens. Histogram of LDA scores of 16S rRNA gene sequences in H9N2 infected and control **a** RIR, and **b** VALO chickens are shown, with a cut-off value of LDA score (log_10_) above 3.0 and *p* < 0.05. Infected groups enriched taxa are indicated with a positive LDA score (green), and taxa enriched in the control groups are characterized by negative score (red)

A mean relative abundance of the dominant bacteria at phyla, class, order, and family levels between H9N2 AIV RIR infected and control chickens (Experiment 1) is shown in Additional file 1: Fig. S5 whereas between RIR and VALO H9N2 infected chickens and its corresponding controls (Experiment 2) is shown in Additional file 1: Fig. S6. Analysis of composition of microbiotas (ANCOM) was applied against group and day of infection variables to determine which bacteria were significantly differentiated in relative abundance at genus level (Fig. 6). The ANCOM results showed significant differences between the control and H9N2 infected RIR groups in members of the *Furmicutes* phylum. Six of *Firmicutes* phylum, *Peptostreptococcaceae* (*Terrisporobacter*), *Planococcaceae* (*Lysinibacillus*), *Erysipelotrichaceae* (*Turicibacter*), *Lachnospiraceae* (*Cellulosilyticum*), *Paenibacillacea* (*Paenibacillus*), *Clostridiaceae* 1 (*Clostridium *sensu stricto 1), were significantly different between the RIR control and H9N2 infected groups and had a high W-statistics and f-score (Fig. 6a). Detailed significant statistics of ANCOM percentile of different taxa is shown in (Additional file 3: Table S5). Furthermore, we also performed Linear discriminant analysis Effect Size (Lefse) analysis based on OTUs to compare the microbial communities between RIR control and RIR H9N2 infected birds at each time point tested. The LEfse analysis and ANCOM generated similar results (Figs. 6 and 7). LEfse results indicated differences in the phylogenetic distributions of the microbiota of H9N2 infected and control chickens at the OTU level (Fig. 7). The gut microbial communities in H9N2 infected birds were different compared to those in control groups. A histogram of the LDA scores was computed for features that showed differential abundance between H9N2 infected and control chickens (Fig. 7a, b). The LDA scores indicated that the relative abundances of *Streptococcaecae* (*Streptococcus*), and *Planococcaceae* (*Lysinibacillus*) were much more enriched in H9N2 infected birds versus control at day 2 post-challenge (Fig. 7a) and the most differentially abundant bacteria taxa (LDA score [log 10] > 3). The most differentially abundant bacterial taxon in control birds was characterized by a preponderance of *Peptostreptococcaceae* (LDA score [log10] > 3) at day 2 post-challenge (Fig. 7a). The differences in the phylogenetic distributions of the microbiotas of H9N2 infected and control chickens at the OTU level were also found at day 4 post-challenge (Fig. 7b). The LDA scores indicated that the relative abundances of *Penicibacillacea (Penicibacillus), Planococcaceae (Lysinibacillus), Erysipelotrichaceae (Turicibacter), Clostridiaceae (Clostridium *sensu stricto* 1)* were much more enriched in H9N2 infected birds as compared to control birds at day 4 post-challenge (Fig. 7b). The control birds were characterized mainly by a preponderance of different *Clostridia* and *Bacillaceae (Bacillus)* (LDA score [log10] > 3). In addition, we saw differential abundance of bacterial taxa between H9N2 infected RIR and VALO and their relative control groups in our second experiment (Fig. 8). The LDA scores indicated that the relative abundances of *Clostridiales (Clostridium *sensu stricto 1) and *Planococcaceae* (Lysinibacillus) (LDA score [log10] > 3.5) were much more enriched in RIR infected birds at day 3 post challenge as compared to the control group, and this correspond to our results obtained in first experiment at day 4 post-challenge (Fig. 7b). In VALO infected chicken, LDA scores indicated that the relative abundances of *Aerococcaceae, Paenibacillaceae, Bacillaceae* (LDA score [log10] > 3.5) were much more enriched at day 3 post-challenge whereas *Clostridiales, Peptostreptococcae and Brevibacteriacea* (LDA score [log10] > 3.5) were significantly reduced in the VALO infected group compared to control group at day 3 post challenge (Fig. 8).

## Discussion

A tremendous number of microorganisms (bacteria, viruses, and fungi), collectively termed the microbiota, are associated with the various host mucosal surfaces and play an important role in host homeostasis [28, 43, 44]. Those microorganisms undergo dynamic changes due to numerous factors, including ageing, changes in diet, environment, or infection by pathogens [28, 45–47]. Recent studies have shown that interaction between host microbiota and viruses may play a crucial role in dictating disease pathogenesis in mammalian hosts [24, 48, 49]. As in all vertebrates, chicken mucosal surfaces are shared by diverse and dynamic population of microbiota [50–52]. In this study, we observed that chicken gut microbiota diversity changes during host maturity, from day 7 post hatch to day 32 post hatch (Fig. 3). The alpha diversity, measured as the number of bacterial taxa, phylogenetic diversity and Shannon index in healthy colon increased over the time of chicken growth in both chicken breeds (RIR and VALO), with most changes being seen in 14 days interval. Temporal increases in the Shannon index suggests a more even bacterial microbiota community evolving with age. Like our findings, Xi et al. [53] showed that major changes in chicken gut microbiota development were observed between day 14 and day 28 post hatch. We found that beta diversity also strongly correlates with time, with the major changes observed in 7 days intervals. Although ageing shaped both alpha and beta diversity in chickens, beta diversity changes occur more rapidly than alpha. The only statistically significant difference in beta diversity of the gut microbiota between RIR and VALO chicken breeds was seen at day 21 of age (Fig. 3).

H9N2 AIV infection in chickens occurs via the respiratory route and the predominant site of initial viral replication is mucosal surface of the oropharyngeal cavity, followed by infection and replication in other sites of the respiratory and intestinal tracts [13, 54]. H9N2 infection in chickens clinically manifests with nonspecific symptoms [55], we observed a mild lethargy and diarrhoea especially between day 2 and day 4 post-challenge in infected chickens of both breeds. Neither breed lost body weight or failed to gain body weight in comparison to the uninfected control groups following H9N2 AIV challenge (Additional file 1: Fig. S1), suggesting that infected chicken consume similar amount of feed as their controls and that this behaviour does not account for the changes in microbiota composition following challenge as it has observed in the influenza infected mice model [56]. Both breeds shed the virus via the oropharyngeal route, but not via cloaca which agrees with other LPAIV infection studies where little, or no shedding was observed from cloacal cavity (Fig. 1) [57–59].

Although we showed in this study that the number of bacterial taxa and Faith's phylogenetic diversity index significantly correlated with time, this correlation was impaired by H9N2 AIV infection. H9N2 infection decreased the number of bacteria taxa during the acute phase of infection (peak viral shedding) and phylogenetic diversity at both acute and recovery phase of infection (Figs. 2 and 4). This suggests that the colon microbiota of H9N2 AIV infected birds lost it overall richness at the acute phase of infection and the microbiota reconstruction appears via increased numbers of predominant bacteria resulting in greater microbiological evenness but not via increased phylogenetic diversity compared to control birds. Zhao et al. (2018) have shown that species richness of faecal microbiota in swans positive for H5N1 AIV infection tended to be lower than that in healthy controls [60]. Furthermore, it was shown that approximately 1,100 of the OTUs identified in the healthy-control swan samples were not detected in AIV H5N1-positive samples [60]. Hird, et al. (2018) have shown that overall species richness in duck species infected with AIV differs within the duck species and for instance only *Anas platyrhynchos* and *Anas carolinensis* showed a significant decrease in alpha diversity in the AIV positive individual [61]. In the current study, we also showed increased Shannon index and phylogenetic diversity in VALO infected chicken as compared to RIR at day 14 post challenge that might suggest different microbiota recovery dynamics occurred between two chicken breeds (Fig. 4). Furthermore, we also observed that beta diversity changes are associated with H9N2 AIV infection at all time points measured in this study (Fig. 5). Like our study, differences in microbiota composition between the control group and H9N2 infected chickens was seen in ileal contents [30], cecum content [29] and faecal swab samples [62] by others. In contrast, Zhao et al. [60] did not notice beta diversity changes in faecal swab samples obtained from migrating whooper swans infected with H5N1 influenza virus. Furthermore, Hird et al. [61] have shown that the microbiota may have a unique relationship with influenza virus infection at the species level. All those findings might suggest that relationship between host microbiota and influenza virus infection might depend on host genetic background of the avian species that is infected by AIV [61] and the strain of infecting AIV. However, these associations require further evaluation.

In this study, it should be noted that the birds in the control and H9N2 infected groups were house in slightly different environments, with control birds being housed in raised floor pens whilst H9N2 infected groups were housed in negatively pressured isolators from 2 weeks of age in the same animal room. Despite this the birds were reared in the same pens and once in the isolators the same quantity and type of food, bedding and enrichment sources were administered to both control and H9N2 infected groups. Our taxonomic analysis showed significant changes in diversity and abundance of the healthy colon chicken followed H9N2 LPAIV infection regardless the chicken line infected (Figs. 7 and 8). Healthy chicken colon in both chicken lines was characterized by predominance of *Proteobacteria* phylum (*Enterobacteriales* order) and *Firmicutes* phylum (*Lactobacillales, Bacillales* and *Clostridiales* orders) and the major differences between H9N2 infected and non-infected chickens were seen in *Firmicutes* phylum. The question why *Bacillales* (*Lysinibacillus*) or *Streptocococeae* are overrepresented during acute phase of influenza infection is open and needs further evaluation.

Futhermore, we observed a dynamic change in chicken colon between acute and recovery phase of AIV infection suggesting that different bacteria taxa might play a role in recovery from infection. Interestingly, phylogenetic abundance distributions of the microbiota in chicken colon differ substantially at day 3 post challenge between RIR and VALO infected chickens (*Clostridiales* in RIR versus *Bacillales* and *Enterobacteriales* in VALO) but was similar at recovery (day 14 post challenge) in both chicken lines, mostly represented by high abundance of *Bacillales* and *Clostridiales* (*Clostriudium *sensu stricto 1). At the same time healthy colon was enriched by *Clostridiales* (*Peptostreptococcae*) in RIR and *Lactobacillales* in both chicken lines. It was previously shown in mice model that *Lactobacillus* spp. have probiotic potential and can improve immune control in influenza infected individuals and thus could aim microbiota recovery following infection [63–65].

## Conclusion

In conclusion, we have shown the dysbiosis in healthy colon microbiota following AIV of H9N2 subtype in two divergent chicken lines. We observed significantly reduced alpha and beta biodiversity during infection. The similarities in dysbiosis of colon microbiota in two chicken lines indicates that influenza virus infection in chickens manipulates the colon microbiota of chickens in a predictable way regardless of breed. *Firmicutes* phylum was the most differentially abundant between infected and non-infected individuals. *Lactobacillales* was missing at recovery phase of infection for both breeds suggesting supplementation of this taxa in during recovery could be beneficial. Although bird breed did not influence the general trend observed in alpha diversity, it did have some impact on beta diversity. Predominance of different bacteria taxa at different time points during influenza infection suggests there is an involvement of chicken gut microbiota. Whilst species specific differences do exist in the response to infection this study supports the idea that microbiota health is important in responding to pathogen infection and that broad population interventions could be utilised in poultry. Modulating the composition of the gut microbiota using probiotics might serve to promote a healthy community that confers protection or mitigates disease from influenza virus infection in chickens.

## Supplementary Information

**Additional file 1.** Supplementary Figures S1–S6.

**Additional file 2.** Suppplementary Tables S1–S4.

**Additional file 3.** Supplementary Table S5.

## Data Availability

The datasets generated and/or analysed during the current study are available in the Figshare repository, https://figshare.com/account/home#/projects/114048.

## Abbreviations

AIV: Avian influenza virus
ANCOME: Analysis composition of microbiotas
CT: Cycle threshold
GMT: Geometric mean titres
HA: Haemagglutinin
HAI: Haemagglutinin inhibition
IBV: Infectious bronchitis virus
LDA: Linear discriminant analysis
LEfSe: Linear discriminant analysis effect size
LPAIV: Low pathogenicity avian influenza virus
MDCK: Mardin–Darby canine kidney
NA: Neuraminidase
NARF: National avian resource facility
NDV: Newcastle disease virus
OP: Oropharyngeal
OTU: Operational taxonomic units
PCA: Principal component analysis
PCoA: Principal coordinate analysis
PFU: Plaque forming units
QIIME: Quantitative insights into microbial ecology
RIR: Rhode island red
SEM: Standard error of the mean
SPF: Specific pathogen free
